# Privacy policies for FDA-authorized or FDA-recognized digital therapeutics impose high digital health literacy demands: a cross-sectional analysis

**DOI:** 10.3389/fpubh.2026.1901627

**Published:** 2026-08-19

**Authors:** Hyun Ji Kim, Hana Moon

**Affiliations:** Department of Family Medicine, Daegu Catholic University School of Medicine, Daegu, Republic of Korea

**Keywords:** digital health literacy, digital therapeutics, document accessibility, health communication, health equity, informed consent, privacy policy, readability

## Abstract

**Background:**

Although digital health literacy is often framed as an individual skill, participation in digitally mediated care is shaped by the demands built into system documents. Inaccessible privacy policies may represent a document-level pathway through which digital health systems contribute to digital exclusion and inequitable participation, particularly for underserved or disadvantaged populations. We examined the accessibility of privacy policies for US FDA-authorized or FDA-recognized digital therapeutics as system-level digital health literacy demands.

**Methods:**

We conducted a cross-sectional document analysis of 36 publicly accessible privacy policies for digital therapeutics with FDA marketing authorization (510(k), *De Novo*, or Premarket Approval) or FDA provisional/discretionary recognition (e.g., enforcement discretion or Breakthrough Device Designation). Accessibility was assessed in two domains: (1) readability burden, summarized as a Composite Grade Mean derived from the Flesch–Kincaid Grade Level, SMOG Index, Gunning Fog Index, and Coleman-Liau Index; and (2) structural and design support, assessed using a prespecified 10-item rubric and summarized as the Structural and Design Support Score (SDSS) compliance rate. Findings were described relative to the prespecified grade 8 benchmark for readability burden and the prespecified 70% SDSS compliance benchmark.

**Results:**

All 36 policies exceeded the grade-8 benchmark. The mean Composite Grade Mean was 16.40 ± 1.60 and the mean Structural and Design Support Score compliance rate was 48.89% ± 11.90%. Only 2 policies (5.6%) met or exceeded the prespecified 70% benchmark. Logical sequencing, descriptive headings, and typographic emphasis were common; internal navigation, key-information summaries, visual aids, and text-visual integration were uncommon. A two-axis mapping analysis showed that 34 of the 36 policies fell within the high readability burden/SDSS <70% quadrant.

**Conclusion:**

On average, privacy policies for FDA-authorized or FDA-recognized digital therapeutics were written above commonly cited public-facing reading-level benchmarks and provided limited structural and design support. These document-level characteristics may plausibly contribute to system-level digital health literacy demands. Improving document accessibility may therefore represent a modifiable target to support more informed participation in digital care and may be conceptually relevant to Sustainable Development Goals 3 (Good Health and Well-being) and 10 (Reduced Inequalities).

## 1 Introduction

Prescription digital therapeutics are now being integrated into clinical care in specific conditions, particularly in behavioral health and addiction care, with emerging real-world evidence in substance and opioid use disorders (1–4). Digital therapeutics (DTx) are defined as health software intended to treat or alleviate a disease, disorder, condition, or injury by generating and delivering a medical intervention with a demonstrable positive therapeutic impact on a patient health (5). From a regulatory perspective, DTx are generally cleared, authorized, or recognized by the FDA under the broader category of Software as a Medical Device rather than as a distinct regulatory class. They encompass software-based interventions intended to deliver a clinical, patient-facing therapeutic effect through repeated use. Because these products pertain to healthcare delivery, their evaluation should extend beyond clinical effectiveness and technical performance to include the conditions that determine whether people can meaningfully access, understand, and engage with digitally mediated care (6, 7). This raises a public health question that extends beyond product efficacy: Are the documentary conditions of access themselves equitable and usable across diverse populations?

In this context, digital health literacy should not be viewed merely as an individual capability (6–8). A broader health literacy perspective suggests that people’s ability to understand and use digital health information depends not only on personal skills but also on how health systems communicate and what demands they impose through services and documents (6, 7). This system-oriented view is especially relevant to public health because inequities may arise from not only users’ capacities but also avoidable communication barriers built into care environments (6–8).

Among the factors that shape participation in digital care, privacy policies warrant particular attention (9–12). These documents typically describe how personal and health-related data are collected, used, shared, stored, and protected, with users often being required to accept them as a condition for using a digital health service or product (9–11). Participation in digital care may therefore depend on not only access to the technology itself but also whether governing documents are understandable and navigable (9). To the extent that such documents are difficult to process, they may create an avoidable barrier at the point where digital care is initiated or sustained (9).

Inaccessible privacy policies may represent a document-level pathway through which digital health systems contribute to digital exclusion and widen inequities in access to digitally mediated care (13, 14). This issue may be particularly relevant for underserved or otherwise disadvantaged populations, for whom documentary burden may intensify existing barriers related to literacy, digital access, and prior experience with complex health systems (13, 15). Improving document accessibility may therefore be conceptually relevant to SDG 3 (Good Health and Well-being) and SDG 10 (Reduced Inequalities) by reducing barriers to participation (16). We present this linkage as a plausible implication rather than as a demonstrated effect because our study did not directly measure equity related outcomes.

This issue can be analyzed through a health literacy perspective that identifies communication problems in not only individual skills but also the demands created by health systems and documents (6–8). Applied to digital health, this perspective suggests that barriers to participation may arise from a mismatch between users’ capabilities and the readability and design demands imposed by the materials through which care is governed (6, 7, 17). In this sense, electronic health (eHealth) literacy remains relevant, but it should be interpreted in relation to the communication environments in which digital care is accessed and used (6, 7, 17).

To evaluate document-level accessibility, we distinguished between two related but noninterchangeable domains: (1) readability burden and (2) structural and design support. The former refers to the level of reading ability required to process the text (18), whereas the latter refers to organizational and visual features that help readers navigate, locate, and interpret information (19, 20). Considering both domains is important because accessibility problems may arise from complex language, weak navigational support, weak visual support, or a combination of these factors (18–20).

Previous research has focused more on user competencies, patient education materials, or the readability of public-facing health information than on the accessibility demands embedded in governance documents such as privacy policies (17, 19–22). Studies of patient education materials and public health webpages have repeatedly revealed substantial readability problems, while work on visual communication has emphasized the role of structure and display in comprehension (19–22). Although prior research has documented readability problems and highlighted the importance of visual and structural support, these strands have rarely been integrated to examine privacy policies as document-level demands within digitally mediated care (9–12).

This study bridges this gap in the literature in two ways. First, it extends digital health literacy beyond an individual-capacity framework by conceptualizing privacy policies as system-level document demands. Second, it operationalizes document accessibility across both readability burden and structural and design support rather than relying on readability formulas alone. Therefore, we conducted a cross-sectional document analysis of publicly accessible privacy policies associated with FDA-authorized or FDA-recognized digital therapeutics, using them as a pragmatic proxy for the documentary conditions potentially shaping entry into digitally mediated care.

## 2 Materials and methods

### 2.1 Study design and conceptual framework

**Figure 1:**
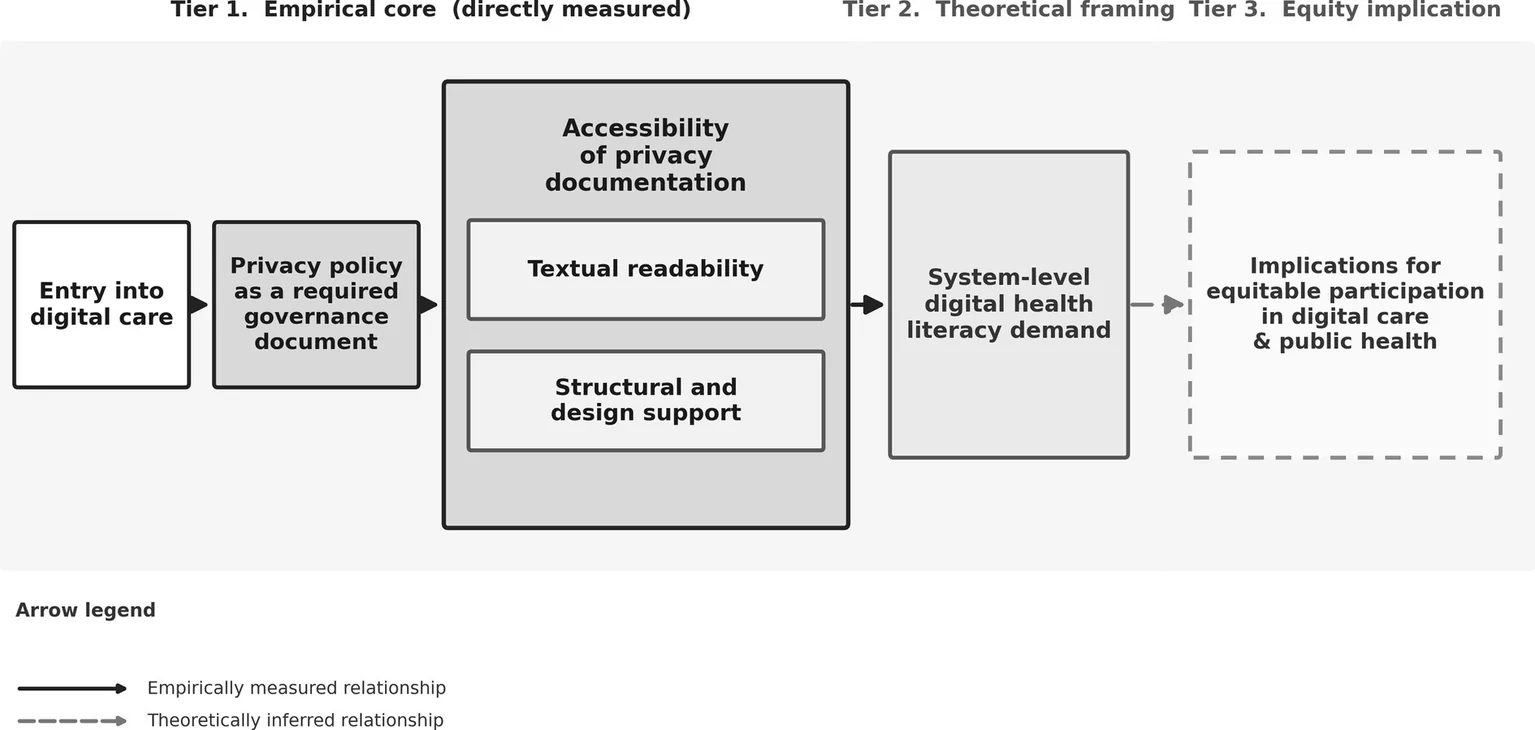
Conceptual model for the document-level privacy-policy accessibility in digital care.

We performed a cross-sectional document analysis of privacy policies associated with digital therapeutics that had received FDA marketing authorization or FDA provisional/discretionary regulatory recognition. The privacy policy was the unit of analysis. The study was informed by a health literacy perspective that considers communication barriers as arising from not only individual skills but also the demands created by health systems, services, and documents (6, 7). On this basis, we treated privacy policies as system-level communication documents potentially influencing whether users can understand and engage with the terms of digital care meaningfully. Figure 1 summarizes the conceptual framework guiding this study.

### 2.2 Operational definition of digital therapeutics

The FDA does not maintain a distinct regulatory category called “digital therapeutic.” Instead, eligible products are cleared, authorized, or recognized under the broader category of Software as a Medical Device, while the term “digital therapeutic” itself is an industry-coined label rather than an FDA regulatory designation. To make the study inclusion criteria explicit and reproducible, we operationally defined digital therapeutics as software-based interventions intended to deliver a clinical, patient-facing therapeutic effect through repeated use and for which the FDA had formally recognized a regulatory status through one of two pathways: (1) formal marketing authorization via the 510(k), *De Novo*, or Premarket Approval (PMA) pathway, or (2) provisional or discretionary regulatory recognition, including products marketed under the FDA’s enforcement discretion policy for low-risk digital health software or products granted Breakthrough Device Designation.

Products were excluded if they lacked therapeutic intent (e.g., general wellness applications without FDA regulatory engagement), consisted of solely hardware devices without an associated patient-facing software intervention or did not involve repeated patient-facing therapeutic use. This definition was independently applied by both authors (HJK, HM), and disagreements regarding borderline cases were resolved through discussion until consensus was reached.

We adopted this broadened definition rather than restricting eligibility to products with formal 510(k), *De Novo*, or PMA marketing authorization alone, because several clinically established digital therapeutics that are widely described in the literature and industry registries as FDA-authorized or FDA-recognized do not hold formal marketing authorization under a narrow interpretation of FDA regulatory pathways. Excluding these products would have yielded a less representative corpus of the digital therapeutics landscape as generally understood by clinicians, developers, and policymakers. Accordingly, this corpus should be interpreted as an operationally defined FDA-engaged therapeutic software sample rather than as a strict regulatory census of all FDA-authorized digital therapeutics.

### 2.3 Sample identification and search strategy

Candidate products were identified through a structured, multisource search strategy rather than a single FDA database query because no single publicly available FDA database comprehensively lists digital therapeutics across all relevant regulatory pathways. Candidate records were compiled from: (1) the FDA 510(k) Premarket Notification database; (2) the FDA *De Novo* Classification database; (3) the FDA’s public guidance documents and communications regarding enforcement discretion for digital health software; (4) the Digital Therapeutics Alliance product registry; (5) company regulatory disclosures (e.g., investor communications and regulatory filings); and (6) industry and trade press coverage of digital therapeutics regulatory milestones. Data collection for sample identification and privacy-policy retrieval was conducted on September 4, 2025.

**Figure 2:**
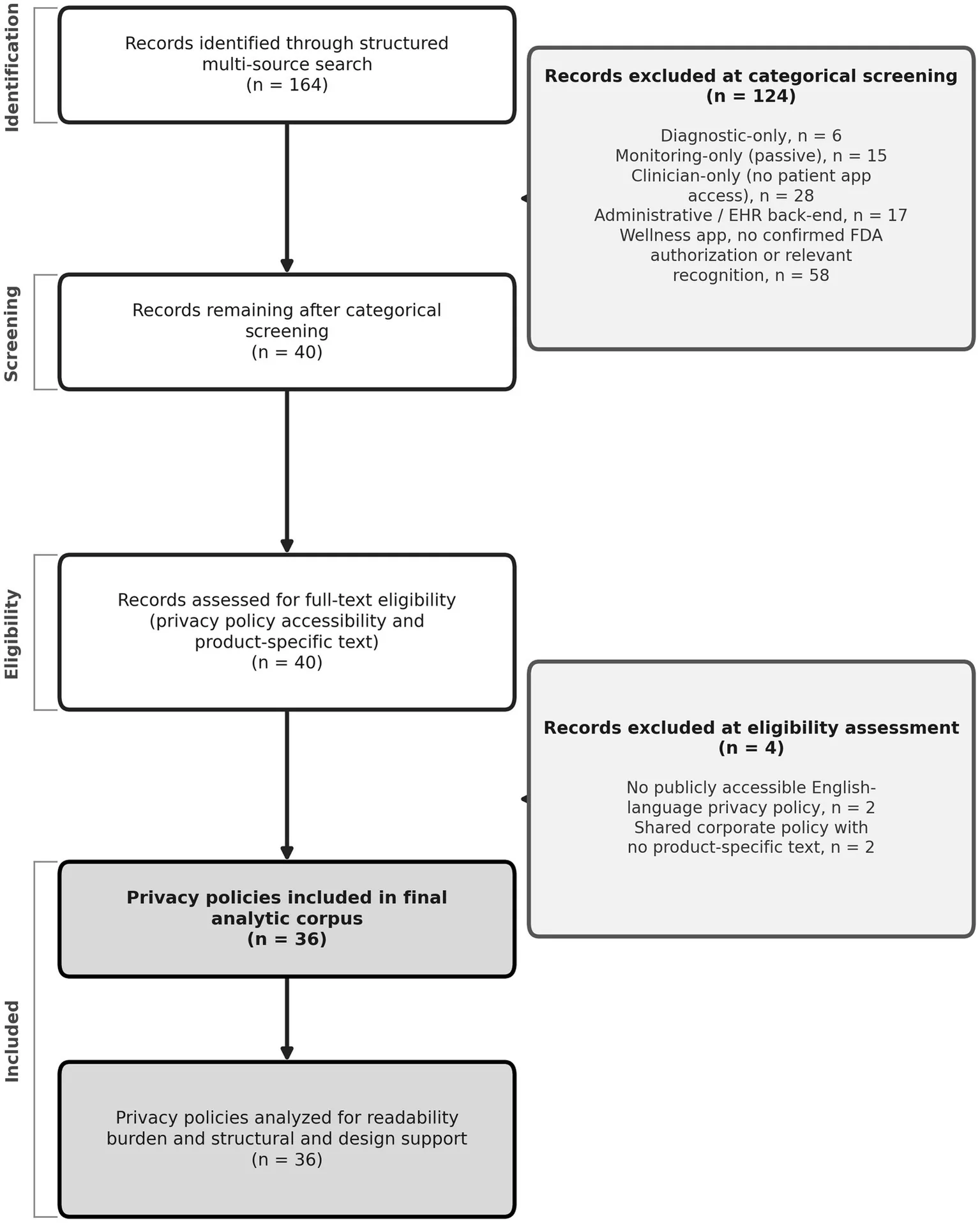
PRISMA-style flow diagram illustrating the identification, screening, eligibility assessment, and inclusion of privacy policies associated with FDA-authorized or FDA-recognized digital therapeutics.

This multisource search strategy initially identified 164 candidate records (Figure 2 and Supplementary material 1). During categorical screening, 124 records were excluded because they did not meet the operational definition above: diagnostic-only software (*n* = 6), passive monitoring-only software without a patient-facing therapeutic function (*n* = 15), clinician-only tools without direct patient app access (*n* = 28), administrative or electronic health record backend software (*n* = 17), and wellness applications without confirmed FDA *De Novo*, 510(k), or Breakthrough Device Designation status (*n* = 58). This process yielded 40 records for full-text eligibility assessment.

### 2.4 Eligibility assessment and document retrieval

At the eligibility stage, all 40 candidate products were to determine whether a publicly accessible, product-specific privacy policy was available. Two records were excluded because no publicly accessible English-language privacy policy was available. Additionally, two duplicate product records were removed because the product pairs (reSET/reSET-O and Sleepio/DaylightRx) shared the same corporate-level privacy policy without product-specific text. This process yielded a final analytic corpus of 36 unique privacy policies (Figure 2 and Supplementary material 1).

For each included product, we recorded the identification source, the authorization or recognition pathway, year of authorization or recognition, developer, privacy-policy URL, and retrieval date (Supplementary material 5). Because privacy policies are living documents that may change over time, we analyzed the most recent publicly accessible version available during the data-collection period rather than the version contemporaneous with the original FDA authorization or recognition. An archived copy of each policy captured at the time of retrieval was retained as the reference document for analysis.

### 2.5 Text preprocessing for readability analysis

For readability analysis, each privacy policy was cleaned according to a prespecified written protocol, converted into plain-text format, and used only for readability computation (Supplementary material 2). The objective of preprocessing was to isolate the substantive privacy policy text while removing webpage interface elements that did not represent policy content and could artificially distort readability estimates. Preprocessing was not intended to simplify, summarize, or otherwise alter the wording, grammar, or legal meaning of the original text.

We independently applied the same written protocol to each policy. Retained content included the main policy body, section headings, legal clauses, and all substantive provisions (e.g., data collection, use, retention, security, user rights, contact information, children’s privacy provisions, and legal definitions). Excluded content was limited to webpage elements unrelated to the substantive policy, including navigation menus, cookie banners, promotional and marketing material, call-to-action buttons, social media links, footer text, copyright notices, search bars, breadcrumb navigation, and duplicated headers or navigation labels. Original sentence and paragraph order were preserved throughout, and sentences were not rewritten, summarized, or simplified.

After independent preprocessing, both investigators compared the cleaned text against the archived original document. We resolved the discrepancies in inclusion or exclusion decisions by consensus before readability computation. The finalized cleaned text was used exclusively for readability analysis. The original unmodified webpage presentation was retained separately for structural and design support coding to preserve layout-dependent features such as headings, internal navigation, typography, and visual organization (Supplementary material 2).

### 2.6 Conceptual domains and measures

As mentioned earlier, we operationalized document-level accessibility in the two complementary rather than interchangeable domains of readability burden and structural and design support. Readability burden was summarized using the Composite Grade Mean, whereas structural and design support was summarized using the Structural and Design Support Score (SDSS) compliance rate.

#### 2.6.1 Readability burden

For the primary readability analysis, we calculated four widely used grade-level readability indices: Flesch–Kincaid Grade Level, Simple Measure of Gobbledygook (SMOG) Index, Gunning Fog Index, and Coleman-Liau Index (23–26). These indices were selected because they are established grade-level readability formulas that provide complementary estimates of reading difficulty.

The primary readability endpoint was the Composite Grade Mean, defined as the arithmetic mean of the four grade-level indices for each privacy policy. It was used to reduce dependence on any single formula and provide a more stable summary estimate of readability burden across policies. We prespecified grade 8 as a pragmatic benchmark used in some health communication and consent-related guidelines (27–29).

#### 2.6.2 Structural and design support

Structural and design support was assessed using a prespecified *de novo* 10-item binary rubric informed by selected domains of the Patient Education Materials Assessment Tool for Printable Materials (PEMAT-P), the PEMAT User’s Guide, and multimedia learning principles (28, 30, 31). Although the rubric was designed to capture document features that may improve navigation, reduce cognitive burden, and support comprehension in public-facing health communication, it was not intended as a validated PEMAT instrument for privacy-policy documents (30, 31).

The 10 items were grouped into the two domains of structural support (Items 1–6), reflecting the logical organization and sequencing of document content, and design support (Items 7–10), reflecting the perceptual and visual presentation of information. This classification was conceptually informed by cognitive load theory, which distinguishes intrinsic from extraneous cognitive load (32, 33). Operational definitions and scoring criteria for each item are presented in Table 1, and illustrative present or absent coding examples for each item are provided in Supplementary material 3 to support consistent rubric application.

**Table 1:** Structural and design support rubric used to assess privacy policy accessibility.

**Domain**	**Item number**	**Item name**	**Definition**	**Cognitive load reduction rationale**
Structural	1	Internal navigation	Presence of a table of contents or internal links enabling direct section access	Reduces extraneous load by eliminating full-document scanning
Structural	2	Key-information summary	A concise summary of key privacy information presented at the document’s beginning	Reduces integration demands across multiple sections
Structural	3	Information chunking	Text divided into short, single-topic paragraphs (≤ 5 sentences)	Limits information processed at one time, reducing working memory burden
Structural	4	Logical sequencing	Information organized in a predictable, logical order	Reduces cognitive effort by aligning with expected document patterns
Structural	5	Actionable instructions	Step-by-step instructions provided for user actions (e.g., opt-out, data deletion)	Reduces inferential effort by converting abstract rights into concrete steps
Structural	6	Descriptive headings	Headings clearly describe each section’s specific content	Guides selective attention and supports efficient section-level navigation
Design	7	Typographic emphasis	Key terms or rights highlighted using formatting such as bold, italics, underlining, or bullet points, excluding headings	Enables rapid identification of important information within dense text
Design	8	Visual aids	Non-textual elements (e.g., icons, diagrams) used to present information	Supports visual processing, reducing reliance on text-only comprehension
Design	9	Text-visual integration	Visual elements functionally explain adjacent text rather than serve decorative purposes	Enhances comprehension through verbal-visual integration (dual coding)
Design	10	Spatial proximity	Visual elements and related text placed in close physical proximity	Reduces split-attention effect by minimizing cross-document search

Each rubric item was coded as present (1) or absent (0). Item scores were summed to generate the Structural and Design Support Score (SDSS; range, 0–10), with higher scores indicating greater structural and design support. The SDSS compliance rate (%) was calculated as (SDSS/10) × 100. Illustrative coding examples for each item are provided in Supplementary material 3.

Each item was coded as present (1) or absent (0), yielding a SDSS ranging from 0 to 10, with higher scores indicating greater structural and design support. We then calculated the SDSS compliance rate (%) as (SDSS/10) × 100.

The items were consistent with the unweighted binary scoring convention used in PEMAT-P (29). They contributed equally to the SDSS because no empirically validated scheme currently exists for assigning differential weights to structural versus design features in privacy policy documents. This equal-weighting approach should be interpreted as a transparent simplifying assumption rather than a claim that all 10 items carry equivalent substantive importance for user comprehension or navigation.

Because no formally validated cutoff exists for structural and design support in privacy-policy documents, we treated 70% as an *a priori*, externally informed benchmark consistent with conventions used in prior PEMAT- and Suitability Assessment of Materials (SAM)-oriented assessment practice, rather than as a validated normative cutoff specific to privacy-policy accessibility (34–37). Accordingly, this benchmark is interpreted descriptively throughout the manuscript rather than as a definitive pass or fail standard.

### 2.7 Reliability procedures

Two raters independently applied the structural and design support rubric to all 36 policies. Before full scoring, both raters reviewed the operational definitions and scored a calibration subset of policies to improve consistency in interpretation. Interrater agreement before consensus adjudication was assessed using Cohen’s *κ* for each binary rubric item and as an overall measure across all 360 rating decisions (36 policies × 10 items), together with overall percent agreement. Discrepancies were resolved through consensus review. Item-level and overall reliability results are reported in Supplementary material 4.

### 2.8 Study objectives and analytic hierarchy

The study had two primary descriptive objectives. The first was to characterize the readability burden of the sample relative to the prespecified grade 8 benchmark. The second was to characterize structural and design support relative to the prespecified 70% benchmark. Secondary analyses described the four individual readability indices, the frequency of each structural and design support item, and the distribution of policies across the two accessibility domains using a descriptive two-axis mapping approach.

### 2.9 Statistical analysis

Text extraction and readability computation were performed in Python (version 3.11; Python Software Foundation) using the textstat and py-readability-metrics libraries. Statistical analyses were performed in R (version 4.3.3; R Foundation for Statistical Computing, Vienna, Austria).

We used descriptive statistics to summarize the readability indices, Composite Grade Mean, SDSS, and SDSS compliance rate, presenting continuous variables as means and standard deviations and categorical variables as counts and percentages. Given the descriptive nature of this document corpus, descriptive statistics rather than inferential hypothesis testing served as the primary basis for our findings. As a supplementary confirmatory analysis, we also compared the mean Composite Grade Mean and the mean SDSS compliance rate with their respective prespecified reference values (grade 8 and 70%). Supplementary confirmatory comparisons with prespecified reference values are reported in Supplementary material 6.

## 3 Ethics statement

Ethics approval and informed consent were waived because this study analyzed publicly accessible documents and did not involve human participants, patient-level data, or identifiable personal information.

## 4 Results

### 4.1 Analytic sample

Using the multi-source search strategy described above, we identified 164 candidate records. Of these, 124 records were excluded for not meeting the operational definition of a digital therapeutic during categorical screening. Eventually, 40 records were assessed for full-text eligibility. During eligibility assessment, four records were excluded (no publicly accessible English-language privacy policy, *n* = 2; shared corporate policy with no product-specific text, *n* = 2), resulting in a final analytic corpus of 36 unique privacy policies (Figure 2 and Supplementary material 1).

### 4.2 Readability burden

**Figure 3:**
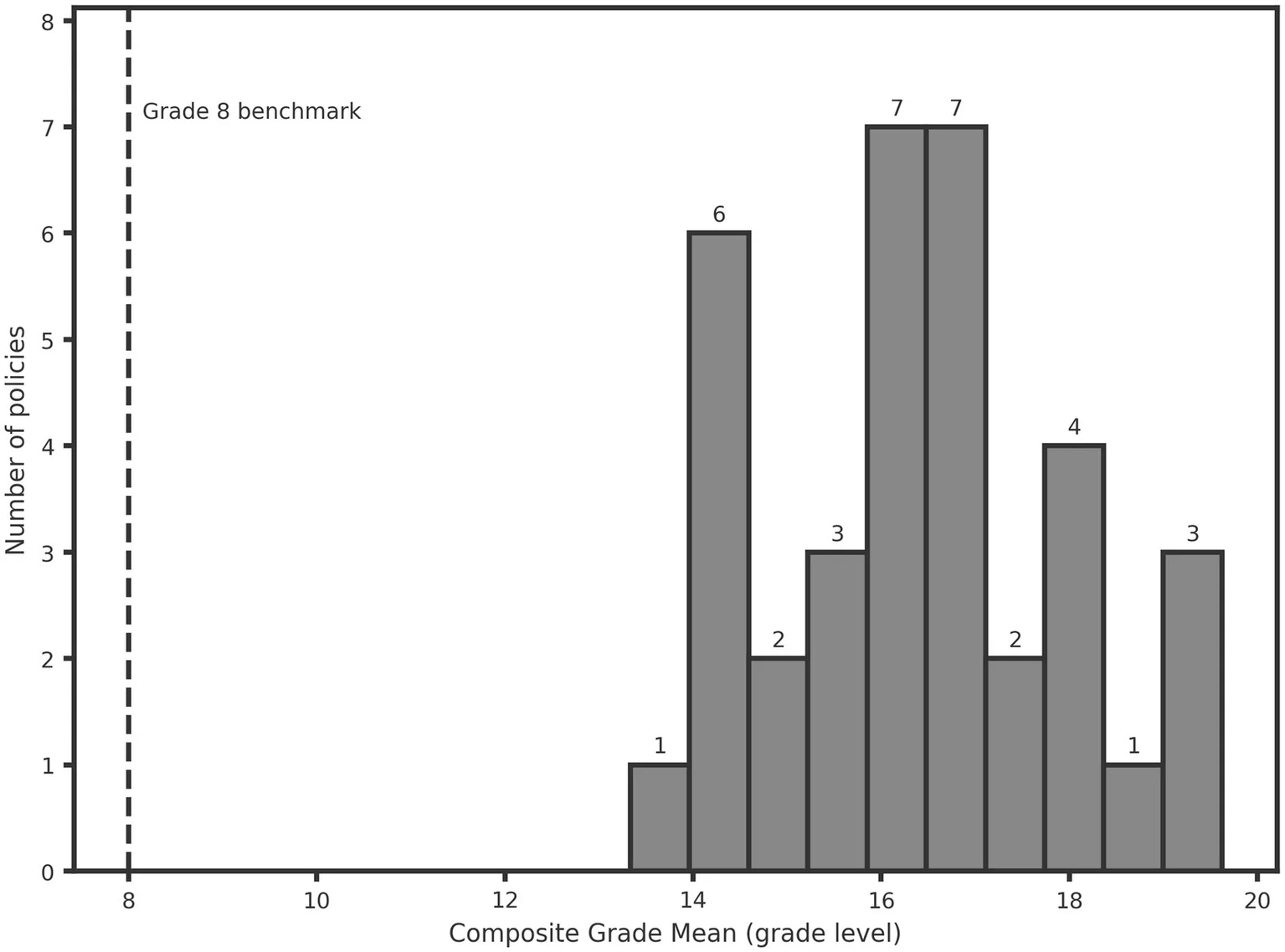
Distribution of readability burden across 36 privacy policies associated with FDA-authorized or FDA-recognized digital therapeutics.

All samples demonstrated high readability burden (Table 2 and Figure 3). The mean Composite Grade Mean was 16.40 ± 1.60 (95% CI, 15.86–16.94; range, 13.33–19.62), and all 36 policies (100.0%) exceeded the prespecified grade 8 benchmark. Supplementary confirmatory analyses supported the same directional conclusion (Supplementary material 6).

**Table 2:** Descriptive summary of readability burden and structural and design support across 36 privacy policies relative to the prespecified grade 8 benchmark and prespecified 70% benchmark.

Outcome	Mean ± SD	95% CI	Range	Benchmark	Description relative to benchmark
(A) Primary outcomes					
Composite Grade Mean	16.40 ± 1.60	15.86–16.94	13.33–19.62	Grade 8	All 36 policies (100.0%) exceeded the benchmark
SDSS compliance rate (%)	48.89 ± 11.90	44.86–52.91	20.00–70.00	70%	2 of 36 policies (5.6%) met or exceeded the prespecified 70% benchmark
(B) Individual readability indices					
Flesch–Kincaid Grade Level	16.34 ± 2.13	15.62–17.06	12.47–20.60	Grade 8	All 36 policies (100.0%) exceeded the benchmark
SMOG Index	17.39 ± 1.45	16.90–17.88	14.93–20.45	Grade 8	All 36 policies (100.0%) exceeded the benchmark
Gunning Fog Index	17.99 ± 2.26	17.23–18.76	13.22–23.00	Grade 8	All 36 policies (100.0%) exceeded the benchmark
Coleman-Liau index	13.89 ± 0.95	13.57–14.21	11.72–16.09	Grade 8	All 36 policies (100.0%) exceeded the benchmark

Values are presented as mean ± SD, 95% confidence interval (CI), and range (minimum–maximum). The sample comprised 36 privacy policies for all rows. The Composite Grade Mean was calculated as the mean of the Flesch–Kincaid Grade Level, SMOG Index, Gunning Fog Index, and Coleman-Liau Index. The SDSS compliance rate (%) was calculated as (SDSS/10) × 100. Given the descriptive nature of this corpus, supplementary confirmatory comparisons against prespecified reference values are reported in Supplementary material 6 and are therefore not reproduced in the present table. Grade-level values should be interpreted as proxies for textual complexity rather than as literal years-of-education requirements or direct measures of comprehension (38). SD, standard deviation; CI, confidence interval; SMOG, Simple Measure of Gobbledygook; SDSS, Structural and Design Support Score.

Mean readability levels were 16.34 ± 2.13 for the Flesch–Kincaid Grade Level, 17.39 ± 1.45 for the SMOG Index, 17.99 ± 2.26 for the Gunning Fog Index, and 13.89 ± 0.95 for the Coleman-Liau Index (Table 2). The Coleman-Liau Index showed modest divergence from the SMOG Index and Gunning Fog Index (Pearson *r* = 0.55–0.72, compared with *r* = 0.93–0.97 among the other three indices), consistent with its reliance on character-based rather than syllable-based complexity cues. This divergence further supported the use of a four-index composite rather than reliance on any single formula. Grade-level values within this range reflect the textual demand captured by these formulas rather than a literal years-of-education requirement. Therefore, these values should be interpreted as proxies for textual complexity rather than as direct evidence of required reading level (38). Policy-level readability burden and structural and design support metrics for all 36 policies are presented in Supplementary material 5.

### 4.3 Structural and design support

Structural and design support was generally limited across the analytic sample. The mean SDSS compliance rate was 48.89% ± 11.90% (95% CI, 44.86–52.91; range, 20.00–70.00), and 2 of 36 policies (5.6%) met or exceeded the prespecified 70% benchmark (Table 2). Supplementary confirmatory analyses yielded the same directional conclusion (Supplementary material 6). These findings indicate that accessibility limitations extended beyond readability burden alone, with most policies combining high readability burden with SDSS values below the prespecified benchmark.

### 4.4 Item-level implementation of structural and design support

**Figure 4:**
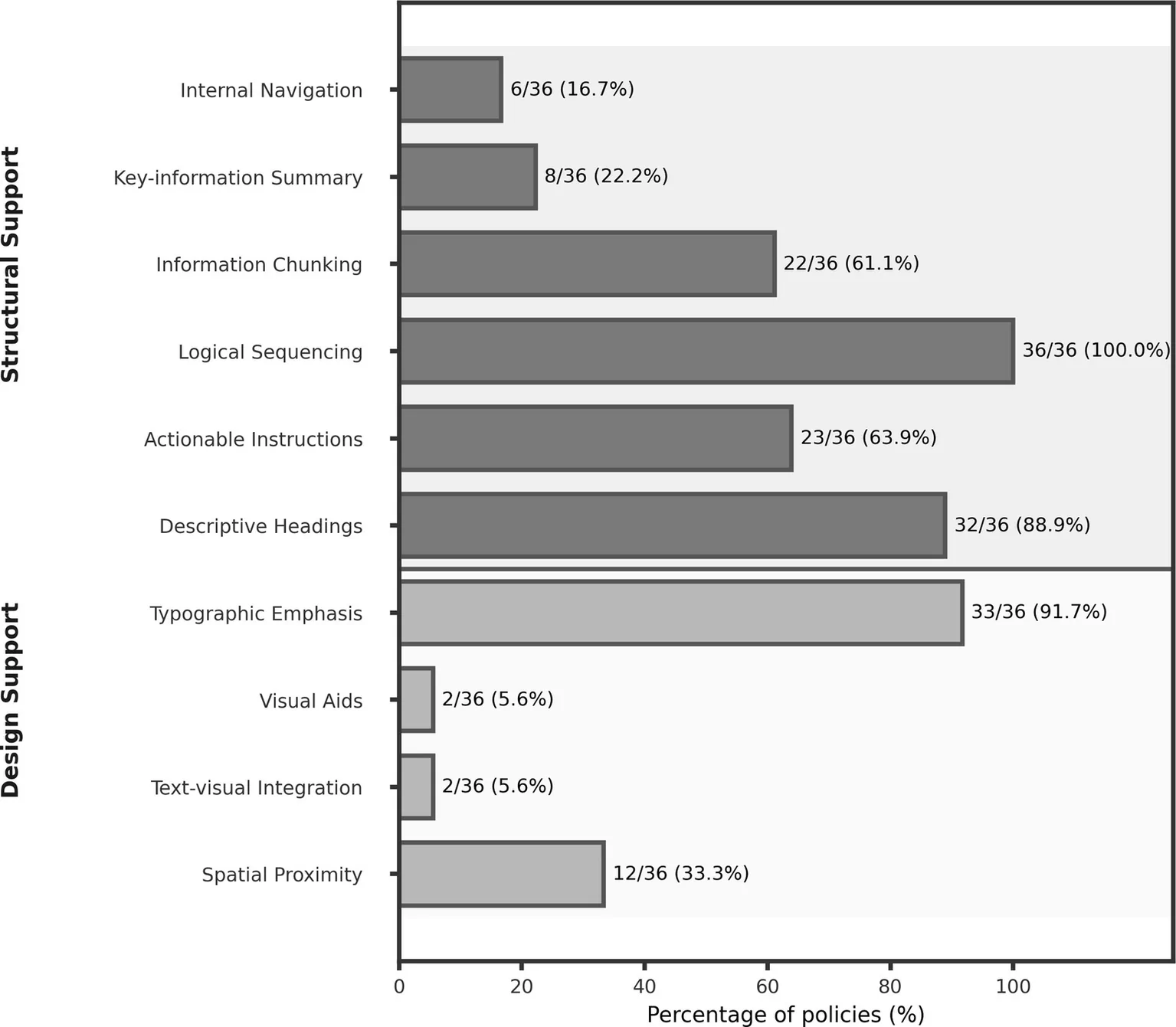
Item-level frequencies of structural and design support features across 36 privacy policies.

Item-level frequencies are depicted in Figure 4. Basic organizational supports, including logical sequencing (100.0%), typographic emphasis (91.7%), and descriptive headings (88.9%), were common. By contrast, features that could reduce navigational effort or support rapid orientation, including spatial proximity (33.3%), key-information summaries (22.2%), internal navigation (16.7%), visual aids (5.6%), and text-visual integration (5.6%), were less common. Actionable instructions (63.9%) and information chunking (61.1%) were present at intermediate frequencies. Overall, basic organizational support was more common than was visual or navigation-enhancing support.

### 4.5 Descriptive two-axis mapping

**Figure 5:**
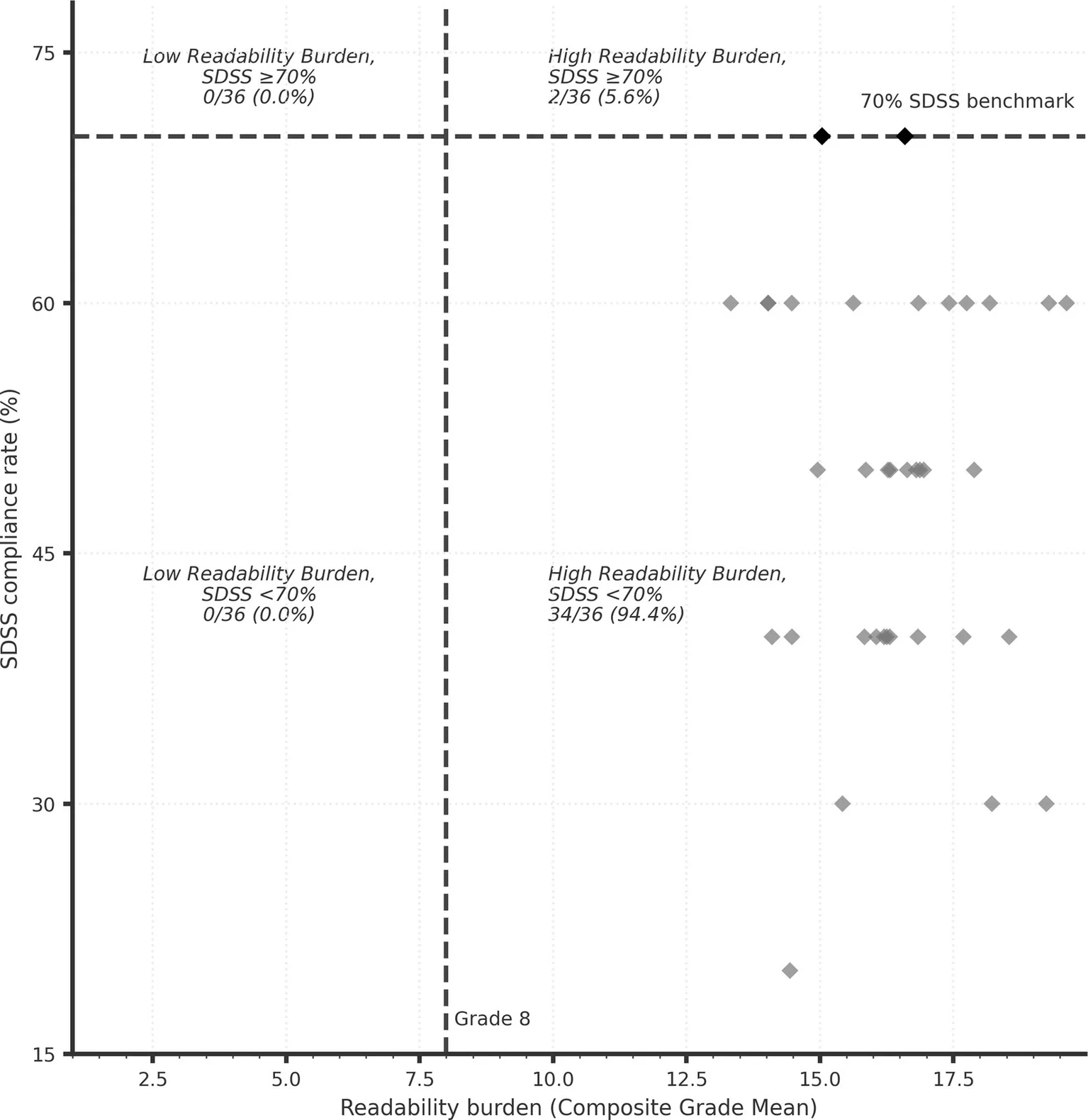
Descriptive two-axis mapping of privacy policies according to readability burden and structural and design support.

Figure 5 maps each privacy policy according to readability burden and SDSS compliance rate. In this descriptive mapping, 34 of the 36 policies (94.4%) fell within the high-readability-burden and below-70% SDSS quadrant, whereas the remaining two policies (5.6%) exceeded 70% on SDSS but still remained above the grade 8 readability benchmark. No policy fell within either of the two low-readability-burden quadrants (Figure 5).

### 4.6 Reliability

Before consensus adjudication, overall interrater agreement across all 360 rating decisions (36 policies × 10 rubric items) was high (Cohen’s *κ* = 0.81; 90.6% agreement; Supplementary material 4). Item-level κ values ranged from 0.43 (moderate) to 1.00 (almost perfect) across nine of the 10 items. For Item 4 (Logical Sequencing), both raters coded the vast majority of policies as meeting the criterion. This produced a high baseline probability of chance agreement. Under this condition, Cohen’s κ approaches zero (κ = 0.00) even when raw percent agreement remained relatively high (77.8%), a well-documented “kappa paradox” for high-prevalence binary ratings (Supplementary material 4).

## 5 Discussion

### 5.1 Overview of principal findings

This cross-sectional analysis of 36 privacy policies associated with FDA-authorized or FDA-recognized digital therapeutics identified a consistent pattern of high readability burden and limited structural and design support. Collectively, these document-level characteristics suggest that the privacy policies in this sample imposed a high readability burden and provided limited structural and design support for navigation and comprehension.

### 5.2 Readability burden interpretation

The readability burden observed in this sample was substantial even in relation to other health communication materials already known to exceed recommended reading levels. Prior studies have revealed that online patient education materials in obstetrics and gynecology and endocrine care are frequently written above levels recommended for the general public, with similar concerns being reported for informed consent forms and research information sheets across multiple clinical settings (38–43). Against this background, the readability levels of the privacy policies in our sample appear demanding for documents that may be encountered before or during engagement with digital care.

This finding is important because privacy policies often explain data collection, data sharing, retention practices, and user rights in dense legal and technical language. Privacy policies that require advanced reading skills may limit users’ ability to understand the terms of participation. In the context of digital therapeutics, where these documents may be encountered before or during treatment engagement, readability burden should be understood not only as a document-quality issue but also as a practical condition for informed participation in care.

These findings should also be interpreted with due consideration of the inherent limitations of formula-based readability estimates (38, 44, 45). Readability formulas rely on selected surface-level text features, and different formulas may yield materially different grade-level estimates for the same document, depending on the metric and analytical procedure applied (44). Accordingly, grade-level values should not be interpreted as indicating the number of years of education required. Moreover, formula-based scores do not directly measure comprehension, perceived difficulty, understandability, or actionability. To reduce reliance on any single formula, we used a composite of four readability indices and interpreted readability burden in conjunction with structural and design support rather than as an independent indicator of users’ likely understanding (44).

### 5.3 Structural and design support interpretation

Insufficient structural and design support further reduced accessibility across the analytic sample. Although basic features such as logical sequencing, descriptive headings, and typographic emphasis were relatively common, features that could help users locate, interpret, and use information more efficiently were often absent—a pattern broadly consistent with prior research showing that patient-facing materials may provide substantial information while still performing unevenly on understandability and actionability (46–50).

Recent studies further suggest that accessibility problems often extend beyond readability alone. Web-based osteosarcoma educational materials, for instance, showed poor readability along with limited understandability and actionability (51), whereas printed lung cancer surgery materials exceeded the recommended readability threshold despite reaching near-adequate scores on understandability, actionability, and accessibility, with substantial variability across institutions (37). Consistent with these findings, a scoping review of low back pain decision aids found generally good understandability but lower actionability and limited use of visual aids (52), while an empirical evaluation of publicly available rabies information found uneven completeness and clarity, particularly for vaccine-specific content (53). In addition, consumer-facing COVID-19 materials produced by state health departments achieved relatively high understandability and actionability, while still leaving room for improvement in readability and clarity (54). Collectively, these studies indicate that document accessibility encompasses both linguistic load and structural-presentational design—consistent with the dual-domain pattern observed in digital therapeutics privacy policies in this study.

### 5.4 Integrated interpretation of the two domains

The main interpretive value of this study lies in the combined assessment of readability burden with structural and design support. Although readability formulas estimate textual difficulty, they do not capture whether a document is easy to navigate, interpret, or use. Prior empirical research has revealed that readability formulas do not fully represent other dimensions of accessibility, including suitability, understandability, actionability, and visual presentation (35, 55, 56). Related studies have also emphasized the importance of document format and visual representation for accessible health communication, including materials designed for people with intellectual disabilities (57). This distinction is important because even a document using simple language may remain difficult to use if its layout, organization, or signaling provide insufficient structural and design support. Conversely, a document with adequate structure may still impose excessive burden if its underlying language is too complex.

Our findings support this integrated view. The privacy policies in this sample were difficult to read, often lacking features that could help users navigate, locate, and interpret key information. The accessibility problem should therefore be understood as a combined documentary burden rather than as a problem of vocabulary alone. This two-domain framework reflects recent efforts to broaden health information assessment through multidimensional and partially automated approaches, including automated multi-feature health literacy tools and systematic multidimensional reviews of patient education materials (56, 58).

### 5.5 Digital exclusion, health equity, underserved populations, and SDG implications

These document-level findings may have implications beyond document design, although this study assessed document characteristics rather than user-level outcomes. Digital exclusion occurs not only when people lack devices, internet access, or technical skills but also when users encounter system documents that are difficult to find, read, interpret, or use. This broader view is consistent with digital health equity scholarship, which frames meaningful access as both connectivity and the capacity to understand and benefit from digital care (13, 14, 59). The documentary burden observed in our sample may plausibly contribute to digital exclusion in digitally mediated care, particularly among users with lower health or digital literacy, limited familiarity with legal and technical documents. However, this relationship was not directly evaluated in the present study.

This issue may be especially relevant for underserved or otherwise disadvantaged populations, including individuals with lower literacy and digital confidence, older age, migration-related barriers, or overlapping forms of social disadvantage. In a migrant community sample, poor eHealth literacy was associated with chronic disease status and reduced capacity to engage with digital health services, illustrating how social position and digital exclusion can intersect at the individual level (60). In this context, inaccessible privacy documentation may plausibly reinforce preexisting barriers to participation in digital care. However, this inference should be regarded as a hypothesis warranting further investigation rather than a demonstrated effect.

These implications are relevant to broader public health goals. Improving privacy policy accessibility may support more informed and usable digital care and is therefore relevant to SDG 3 (Good Health and Well-being) and SDG 10 (Reduced Inequalities). These considerations may be salient in the emerging prescription digital therapeutics literature, where concerns have been raised about methodological rigor, inclusivity, and policy-related barriers to access (61–63). However, because this study did not directly measure comprehension, trust, uptake, or health outcomes, the equity and SDG framing should be interpreted as a plausible implication rather than as a demonstrated pathway.

### 5.6 Methodological contribution

Previous research on digital health privacy policies has documented recurring problems not only in readability, but also in policy availability, app-specific scope, transparency, and ease of navigation across general mHealth ecosystems and several subdomains, including mental health, menstruation-tracking, contact-tracing, and AI-enabled applications (9–12, 64–67). Our findings align with this broader literature, but extend it by examining a more clearly bounded regulatory corpus of FDA-authorized or FDA-recognized digital therapeutics. Accordingly, the present study does not suggest that digital therapeutics are uniquely problematic. We demonstrated that accessibility problems were already reported across digital health privacy documents. They remain present even in a clinically oriented and comparatively regulated segment of the market.

This dual-domain approach provides a more comprehensive description of document accessibility than readability formulas alone. It also offers a practical framework for future monitoring of whether document revisions reduce literacy demand across both textual and structural dimensions. Recent work on automated health literacy assessment suggests that multidimensional evaluations of written health information may become increasingly scalable as multi-feature tools mature (58). Therefore, the present study provides not only empirical findings but also a reproducible framework to assess document-level accessibility in regulated digital health settings.

At the same time, privacy policies are not written solely as patient education materials. They also function as legal and compliance documents (68). Their wording may therefore be shaped by disclosure obligations, risk-management concerns, third-party data-sharing arrangements, and uncertainty regarding future data use within evolving digital ecosystems. This context does not excuse poor accessibility, but it helps explain why simplification is not always straightforward. Major privacy frameworks also emphasize that privacy information should be communicated using clear, plain, and accessible language (69, 70). The practical goal, therefore, is not legal completeness at the expense of accessibility, but legal completeness delivered through more accessible communication design and drafting (68, 70). Accordingly, our findings should be interpreted as evidence of documentary complexity and limited navigational support rather than as direct evidence of patient misunderstanding, mistrust, or downstream inequity.

### 5.7 Strengths

This study has several strengths. First, it examined a clearly defined, policy-relevant corpus of privacy policies associated with FDA-authorized or FDA-recognized digital therapeutics, identified through an explicit, multisource search strategy and a transparent operational definition, rather than a heterogeneous set of general health apps or websites. Second, it reduced dependence on any single readability formula by using a composite measure derived from four widely used indices. Third, it assessed document accessibility across two complementary domains—textual readability and structural and design support—thereby providing a more comprehensive account of documentary burden than text-only approaches. Fourth, using prespecified benchmarks for readability burden and SDSS compliance rate improved the interpretability and policy relevance of the findings. Finally, the item-level assessment helped identify specific and actionable targets for document improvement, such as key-information summaries, internal navigation tools, and visual supports. Collectively, these features strengthen the descriptive, methodological, and public health relevance of the study.

### 5.8 Limitations

This study has several limitations. First, because it involved a document-based analysis, it did not directly test whether the observed document characteristics were associated with user comprehension, trust, informed decision-making, uptake, sustained use, or downstream equity outcomes; the findings should be interpreted as evidence of documentary burden rather than as direct evidence of behavioral or health effects. Second, the analytic sample was restricted to publicly accessible privacy policies associated with digital therapeutics meeting our operational definition, which limits generalizability to other digital health products or to policies delivered only within app-based, clinical, or commercial workflows. Third, although the composite readability approach improves robustness relative to any single formula, readability formulas remain proxy measures of textual difficulty and do not capture actual comprehension. This limitation is particularly important because privacy policies often contain dense legal terminology, proper nouns, and enumerated clauses that may inflate grade-level scores independent of true reading difficulty. We also did not perform a formal sensitivity analysis comparing alternative preprocessing rules. Therefore, we cannot exclude the possibility that small inclusion or exclusion decisions may have affected some readability estimates. Fourth, the structural and design support rubric was adapted from PEMAT-P rather than independently validated for privacy-policy documents. The rubric has not undergone formal psychometric validation (e.g., confirmatory factor analysis, test–retest reliability, criterion validity), and the prespecified 70% benchmark should therefore be interpreted as a descriptive benchmark rather than a validated normative cutoff. Fifth, the rubric could not represent all aspects of usability, visual hierarchy, or interactive reading experience. Sixth, privacy policies are also shaped by mandatory legal disclosure requirements, including those under the Health Insurance Portability and Accountability Act and the General Data Protection Regulation (68, 70). This study could not fully separate avoidable drafting choices from content that legal requirements may have constrained. Finally, because the study was cross-sectional, revision history or temporal improvement in document accessibility could not be assessed.

## 6 Conclusion and policy implications

Privacy policies associated with FDA-authorized or FDA-recognized digital therapeutics, as written during our data-collection period, were generally above commonly cited public-facing readability benchmarks and provided limited structural and design support. In this sample, the documentary burden was not limited to complex language but also reflected limited structural and design support, with few policies meeting the prespecified 70% benchmark. These findings suggest that privacy-policy accessibility may be a modifiable feature of digital care infrastructure, worth attention alongside other documentation-quality issues. Practical improvements could include plain-language revision, layered summaries, clearer headings, internal navigation tools, and stronger visual and layout-based supports. Although such changes would not eliminate broader structural inequalities in digital health access, they may plausibly reduce one avoidable source of documentary burden and support more equitable participation in digitally mediated care. However, confirming this potential benefit will require studies that directly measure user comprehension, trust, and engagement.

## Data Availability

The original contributions presented in the study are included in the article/Supplementary material, further inquiries can be directed to the corresponding author/s.
